# Repeatability and reproducibility of brain age estimates in multiple sclerosis for three publicly available models

**DOI:** 10.1016/j.ynirp.2025.100252

**Published:** 2025-03-21

**Authors:** Lonneke Bos, David R. van Nederpelt, J.H. Cole, E.M.M. Strijbis, B. Moraal, J.P.A. Kuijer, B.M.J. Uitdehaag, F. Barkhof, A.M. Wink, H. Vrenken, B. Jasperse

**Affiliations:** a MS Center Amsterdam, Radiology and Nuclear Medicine, Amsterdam Neuroscience, Amsterdam UMC Location VUmc, Amsterdam, the Netherlands; b UCL London, Institutes of Neurology and Healthcare Engineering, London, United Kingdom; c MS Center Amsterdam, Neurology, Amsterdam Neuroscience, Amsterdam UMC Location VUmc, Amsterdam, the Netherlands

**Keywords:** Brain age, Multiple sclerosis, Reliability

## Abstract

Accelerated brain aging is a marker of disease-related neurodegeneration in multiple sclerosis (MS). Artificial intelligence models, trained on healthy individuals, can estimate age from brain MRI scans, but the effects of technical variations between MR scanners and conditions on these estimates are currently insufficiently investigated. This study aims to determine the within-scanner repeatability and between-scanner reproducibility of the brain-predicted age difference (brain-PAD) across three brain age models.

30 people with multiple sclerosis and 10 healthy controls (mean age 44.2 ± 11.7 years and 39.2 ± 12.9 years, respectively), underwent six scans in a single day; a scan and immediate on a 3 T GE, 1.5 T Siemens and a 3 T Siemens MRI-scanner. Brain-PAD was determined using brainageR, DeepBrainNet and the MIDI-model from 3D T1w brain MRI-scans. Intraclass correlation coefficient (ICC) was used to quantify absolute agreement within-scanner (ICC-AA) and between-scanner consistency (ICC-C). Variance component analyses were used to determine the standard error of measurement (SEM) and the smallest detectable change (SDC).

Brain-PAD was higher for pwMS compared to HC when predicted with brainageR and DeepBrainNet, not when predicted with the MIDI-model. Within-scanner repeatability was excellent (ICC-AA>0.93) for all models. Between-scanner reproducibility was good to excellent (ICC-C>0.85) for brainageR and the MIDI-model, while DeepBrainNet, showed excellent between-scanner reproducibility for Sola vs. VIDA (ICC-C:0.97), but moderate for GE vs. Sola and for GE vs. Vida (ICC-C:0.63 and 0.61). Between-scanner SDC was 6.56 years for brainageR, 5.57 years for the MIDI-model and 22.65 years for DeepBrainNet.

Our findings demonstrated high repeatability of brain age estimates from the same scanner, but variable reproducibility across different scanners, irrespective of the brain age prediction model.

## 1 Introduction

Multiple sclerosis (MS) is a demyelinating disease characterized by inflammation and neurodegeneration in the central nervous system (Compston et al., 2008). Neurodegenerative processes are partly responsible for the development of clinical disability, and therefore require monitoring to make well informed treatment decisions (Liu et al., 2018; Bjartmar et al., 2001). The macroscopic structural changes of the aging brain are similar to neurodegenerative brain changes observed in various brain diseases, including MS (Cole et al., 2018). Apparent accelerated aging of the brain in MS can therefore be considered as a marker of neurodegeneration.

Recent machine and deep learning techniques, trained on healthy aging individuals, can estimate a person's age from conventional brain magnetic resonance imaging (MRI) scans. By subtracting the person's chronological age from this estimated brain age, accelerated aging of the brain can be expressed, and the brain predicted age difference (brain-PAD) can be obtained. Brain-PAD was found to be increased by 6–12 years in people with MS, related to clinical and radiological evidence of disease severity in MS (Cole et al., 2020; Høgestøl et al., 2019; Kaufmann et al., 2019) and predictive of future clinical disability (Cole et al., 2019). Considering that brain-PAD takes changes in the entire brain into account, and its intuitiveness, makes it a promising alternative for depicting disease-related neurodegeneration.

A variety of brain age prediction models have been developed. The performance of these models has mainly been evaluated on brain MRI scans acquired on the same machine in a single session (Bacas et al., 2023). Technical variations in brain age estimation can occur *between* scanners due to differences in acquisition protocols (Van Nederpelt et al., 2023), vendors and field strengths (Amiri et al., 2018), but also *within* scanners due to, among others, thermal noise and patient positioning. We define the consistency of measurements *within*-scanner as *repeatability*, whereas we define consistency of measurements *between*-scanners as *reproducibility*. To thoroughly determine the effect of these scanner-related variations on within-scanner repeatability and between-scanner reproducibility of brain age estimates, repeated acquisition of brain MRI scans on different MRI machines performed over a short time interval are required.

Systematic assessment of how these scanner-related variations affect brain age estimates is crucial for the implementation of brain age models in research and clinical settings. Therefore, the aim of this study is to determine within scanner repeatability and between scanner reproducibility of brain age estimates by three publicly available models in people with MS (pwMS) and healthy controls (HC) that were scanned two times on three different MRI scanners within one day.

## 2 Materials and methods

### 2.1 Participants

30 people with MS (pwMS) and 10 healthy controls (HCs) were included from the “accurate multiple sclerosis atrophy measurement system” (AMS2) dataset (Van Nederpelt et al., 2024a). Inclusion criteria were aged between 18 and 70 years, and being able and willing to undergo 6 MRI scans in one day. PwMS had a clinically definite relapsing remitting MS (RRMS), secondary progressive MS (SPMS) or primary progressive MS (PPMS) diagnosis according to the 2017 revised McDonald criteria (Thompson et al., 2018). Exclusion criteria included any clinically relevant neurological, auto immune, or neuropsychological comorbidity other than MS and contraindication to undergo MRI examination. The institutional review board approved the study protocol (NL75420.029.20) and written informed consent was obtained from all individuals, according to the Declaration of Helsinki.

### 2.2 MRI protocol

All participants were scanned twice on each of the following scanners: 1) 3 T GE Discovery MR750 with an 8-channel phased array head coil (GE Healthcare, Milwaukee, USA), 2) 3 T Siemens Vida with a 20-channel head-neck coil (Siemens Healthineers, Erlangen, Germany) and 3) 1.5 T Siemens Sola with a 20-channel head-neck coil (Siemens Healthineers, Erlangen, Germany) in random order. At each scanner, the participants underwent a scan and rescan, hereafter referred to as first (scan) and second (rescan) run. After each first run, the patient was removed from the scanner bed, walked a few steps, and repositioned in the scanner for the second scan. This procedure was repeated on each scanner, resulting in six acquisitions per patient. All MRI acquisitions were performed within 4 h from the start of the first acquisition. All exams were scanned by the same trained individual (DvN). When visually obvious motion was detected during acquisition, the scan was repeated immediately. Table 1 summarizes the acquisition parameters for the 3D T1w sequences used in this study.

**Table 1:** MRI acquisition parameters for the T1w sequences used in this study, TR = Repetition Time; TE = Echo Time; TI = Inversion Time; FSPGR = Fast SPoiled GRadient Echo; MPRAGE = Magnetization Prepared - RApid Gradient Echo.

Scanner	Pulse sequence name	Resolution (mm^3^)	TR (ms)	TE (ms)	TI (ms)	FA (°)
GE Discovery MR750	FSPGR	*1.0 × 1.0 × 1.0*	8.2	3.2	450	12
Siemens Sola	MPRAGE	*1.0 × 1.0 × 1.0*	2300	2.6	900	8
Siemens Vida	MPRAGE	*1.0 × 1.0 × 1.0*	2300	2.3	900	8

### 2.3 Brain age models

For the brain age estimation and subsequent brain-PAD calculation, three widely used brain age algorithms were used on our dataset: brainageR (Clausen et al., 2022; Hobday et al., 2022; Biondo et al., 2022), DeepBrainNet (Bashyam et al., 2020) and the MIDI-model (Wood et al., 2022). The brain age algorithms were all developed using a large sample size in the training dataset (>2000 participants), had a Pearson's correlation between chronological age and brain-predicted age of r > 0.960, and they varied in their predictive properties for brain age (see Table 2). The brain age models were trained on T1w scans, and each training dataset consisted of various scanners.

**Table 2:** Overview of the properties of brainageR, DeepBrainNet and the MIDI-model. GPR = Gaussian Process Regression, MNI = Montreal Neurological Institute.

Brain age model	Network properties	Pre-processing	Type of training data	# training data	Mean absolute error (years)
brainageR	GPR of volumes	Skull-stripping, registration to MNI space, bias field correction^a^	T1w	3377	4.9
DeepBrainNet	2D Inception-resnetv2	Skull-stripping, registration to MNI space	T1w	11,729	3.7
MIDI-model	3D DenseNet121	Skull-stripping	T1w + T2w	2387	3.4

a Pre-processing steps are performed internally by SPM12.

#### 2.3.1 brainageR brain age model

Pre-processing in brainageR consists of bias field correction and registration to standard space (MNI152) T1w brain MR images and segments gray matter, white matter, CSF using statistical parametric mapping software (SPM12) (Ashburner et al., 2014). Resulting segmentations are then converted to principal components and used to predict a previously trained Gaussian process regression (implemented in R, kernlab package (Karatzoglou et al., 2019)) model to estimate brain age. The model was trained on n = 3,377 healthy individuals (mean age ± SD (range) = 40.6 ± 21.4 (18–92) years) from seven publicly available datasets. The reported mean absolute error (MAE) for this model was 4.9 years. Code is available at: https://github.com/james-cole/brainageR.

#### 2.3.2 DeepBrainNet brain age model

DeepBrainNet is a 2D convolutional neural network (CNN) which uses the inception-resnetv2 framework (Bashyam et al., 2020). Brain age is determined on T1w images of the brain after skull-stripping with HD-BET (Isensee et al., 2019) and affinely registering to MNI-space with FMRIB's Linear Image Registration Tool (FSL-FLIRT (Jenkinson et al., 2002; Jenkinson and Smith, 2001)) with default options. The model then independently predicts brain age for each of the 80 axial slices of the brain MRI. The final brain age estimate is the median of these 80 brain age estimates. DeepBrainNet was trained on n = 11,729 healthy individuals (range = 3–95 years old) from 18 different datasets. The authors reported a MAE for this model of 3.7 years. Relevant code is available at: https://github.com/vishnubashyam/DeepBrainNet.

#### 2.3.3 MIDI brain age model

The MIDI-model is based on the DenseNet121 architecture (Wood et al., 2022). The model predicts brain age on T1w skull-stripped images using HD-BET (Isensee et al., 2019). The model was trained as an “ensemble” model which was a combination of raw volumetric T1w and axial T2w images, but only requires T1w images to predict brain age. The model is designed to estimate brain age from 3D MRI scans by extracting features through convolutional blocks, transitioning layers, and global average pooling (Cardoso et al., 2022). The ensemble model was trained on n = 2,387 healthy individuals (mean age ± SD (range) = 32.8 years ± 12.3 (18–87)). The reported MAE for this model was 3.4 years. Relevant code is available at: https://github.com/MIDIconsortium/BrainAge.

### 2.4 Statistical analyses

Statistical analyses were performed using R Statistical Software (version 4.1.1; R Foundation for Statistical Computing, Vienna, Austria). To evaluate for differences in brain-PAD between healthy controls and pwMS, a linear regression model was used, corrected for sex. The effect size to differentiate between HC and pwMS was calculated using Cohen's d, which is calculated as the difference between the means of the two groups divided by the pooled standard deviation (Sullivan and Feinn, 2012; Fritz et al., 2012). Because the purpose of this study was not to assess accuracy, MAE's based on the 10 HC in this study are reported in the Supplementary Table 1.

#### 2.4.1 Repeatability and reproducibility

The repeatability and the reproducibility of the brain-PAD estimates were assessed with the intraclass correlation coefficient (ICC) for absolute agreement (ICC-AA) within scanner and, for consistency (ICC-C), between scanners (Van Nederpelt et al., 2023). The ICC-AA assesses exact agreement; the ICC-C disregards systematic difference between measurements. The ICC outcomes were interpreted using the standards of Koo and Li (2016). The between scanner ICC-C was determined on brain-PAD of the first runs. Stratified ICC analysis for HC and pwMS was conducted to assess repeatability and reproducibility on group level. Additionally, to quantify differences between the models, the between model ICC-C was determined on the brain-PAD of the first runs. Systematic differences were assessed with repeated measures ANOVA (RM-ANOVA). When appropriate, this was followed by post-hoc testing using the Wilcoxon signed rank test. Significance was set at *p* < 0.05.

#### 2.4.2 Precision

Precision in this study was defined as the consistency of brain-PAD measurements. Bland-Altman plots were created to assess any systemic bias and the limits of agreement of the brain-PAD measurements. These plots depict the difference of brain-PAD as a function of the average of brain-PAD with accompanying 95% confidence intervals. To assess measurement precision, we performed a variance component analysis and computed the standard error of measurements (SEM) for within-scanner (SEM_within_) and between-scanner (SEM_between_) measurements. Here, the SEM_within_ was defined as the square root of the residual variance (σ^2^_ε_): ${SEM}_{within}=\sqrt{\sigma_{\varepsilon }^{2}}$, and the SEM_between_ was defined as the square root of the sum of the scanner variance (σ^2^_s_) and σ^2^_ε_: $SEMbetween=\sqrt{\sigma_{s}^{2}+\sigma_{\varepsilon }^{2}}$. Finally, the smallest detectable change was calculated to quantify the minimal change needed to confidently identify a true difference, beyond what could be attributed to measurement error (De Vet et al., 2011; Mokkink et al., 2023). The SDC was derived from the SEM, as SDC = 1.96 · $\sqrt{2}$ · SEM.

## 3 Results

### 3.1 Descriptives

**Fig. 1:**
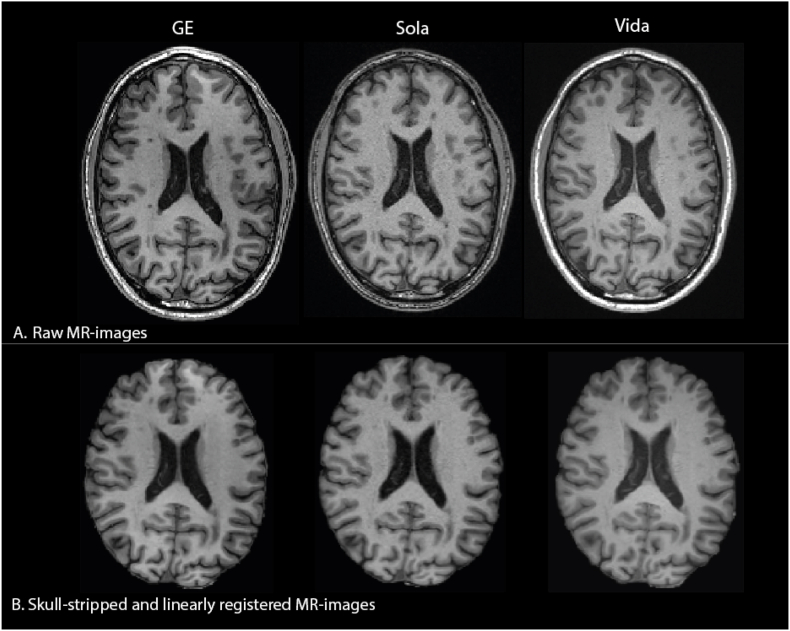
**A***Example of the raw T1w MR images of a pwMS (male, age* = *57.8 years, disease duration* = *2.9 years, EDSS* = *2, phenotype* = *RRMS).****B****Pre-processed derivatives of****A****after skull-stripping (HD-BET) and linear registration to standard MNI space (FSL-FLIRT).*

**Fig. 2:**
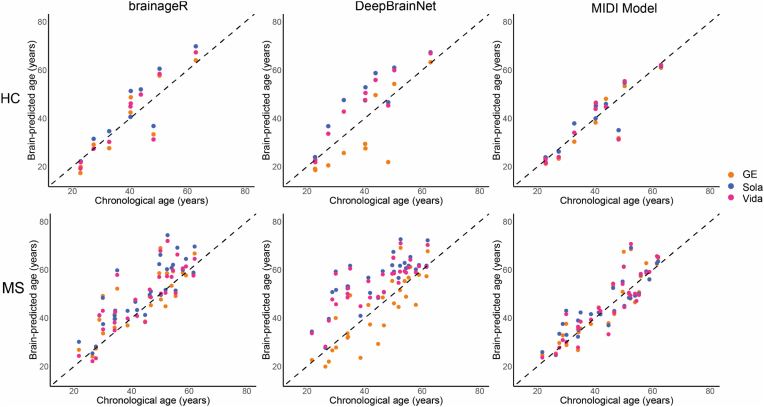
Scatterplot showing the relationship between chronological age (x-axis) and brain-predicted age (y-axis) for healthy controls (top row) and pwMS (bottom row) for each of the three models. Only the first run is shown. The dotted line represents the identity line.

Fig. 1 shows examples of the original and pre-processed T1w images obtained from each scanner. The 30 pwMS had a mean age of 44.4 years (SD:11.66) and a mean EDSS of 3.3 (SD: 1.9) with a mean disease duration of 7.4 years (SD:6.5) (range: 0.7–23.7 years) (Table 3). No differences were observed between pwMS and HC regarding age or gender. Fig. 2 shows the predicted brain age in years, versus the chronological age for both HCs and pwMS for all three models. Healthy controls deviate less from the identity line, compared to pwMS. For DeepBrainNet, brain age estimates with GE are consistently below the Sola and Vida for brainageR and the MIDI-model, the brain age estimates are more similar between scanners.

**Table 3:** Demographics and clinical characteristics, EDSS = Expanded Disability Status Scale; MS = multiple sclerosis.^a^ Mean,^b^ median. There were no differences for age between healthy controls (HC) and people with MS (pwMS).

	HC	pwMS
n	10	30

Subject type (RRMS/SPMS/PPMS)		22/2/6
Female, n (%)	7 (70)	20 (67)
Age^a^, years ± SD (range)	39.17 ± 12.93 (22.8–62.9)	44.4 ± 11.7 (21.7–61.9)
EDSS^a^ ± SD (range)		3.3 ± 1.9 (0–6.5)
Disease duration^a^, years ± SD (range)		7.4 ± 6.5 (0.7–23.7)
Lesion volume^b^, mL (range)		5.9 (1.7–51.2)

### 3.2 Group and scanner differences

**Fig. 3:**
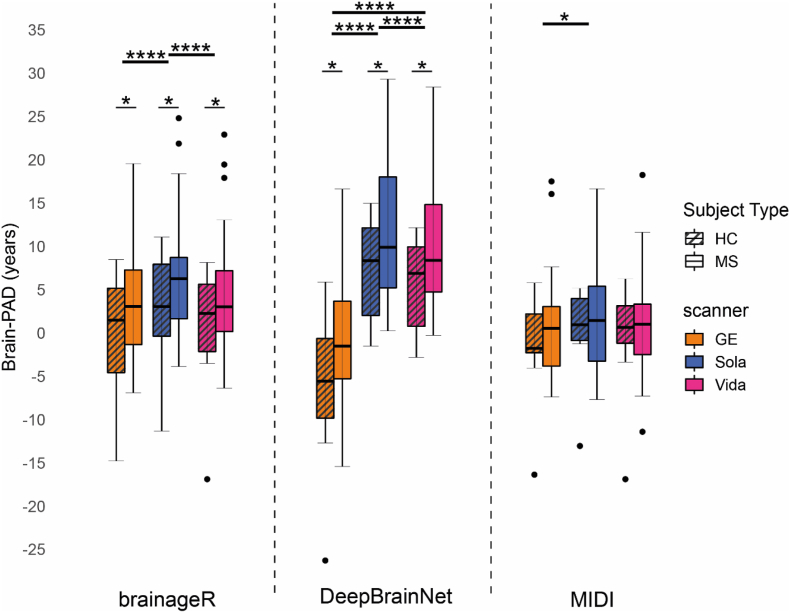
Boxplots of brain-PAD estimates for the three brain age models, the first scan of the three scanners for healthy controls and pwMS. Boxplots (Tukey, line at median) of the brain-PAD measurements for brainageR, DeepBrainNet and MIDI-model for three scanners, respectively. A Mann-Whitney *U* test was used to evaluate differences between pwMS for each model. The differences between HC and pwMS for each scanner and model were evaluated corrected for sex. Within each model, the differences between the scanners were analyzed with a repeated measures ANOVA, followed by post-hoc testing with the Wilcoxon signed rank test. ∗p < 0.05, ∗∗p < 0.01, ∗∗∗∗p < 0.0001.

Brain-PAD was higher for pwMS compared to HC for brainageR (mean difference ± standard deviation; GE: +4.05 ± 1.88 years; Sola: +4.30 ± 1.76 years; Vida: +4.11 ± 1.84 years) and DeepBrainNet (GE: +5.22 ± 2.14 years; Sola: +4.49 ± 1.86 years; Vida: +4.49 ± 1.73 years) (Fig. 3). The MIDI-model did not show differences between these two groups (GE: +2.31 ± 1.56 years; Sola: +1.85 ± 1.44 years; Vida: +1.57 ± 1.60 years). The effect size (Cohen's d) for brain-PAD differences between HC and pwMS is medium for brainageR (between −0.57 and −0.53 for all scanners), medium for DeepBrainNet (between −0.57 and −0.53 for all scanners), and small for the MIDI-model (between −0.38 and −0.24 for all scanners).

The RM-ANOVA showed differences of brain-PAD between the three scanners for brainageR, DeepBrainNet, and the MIDI model, respectively. Post-hoc analysis for brainageR with the Wilcoxon signed rank test showed that brain-PAD derived from Sola scans was significantly lower compared to GE and Vida. Post-hoc analysis of DeepBrainNet showed differences between all scanners. Post-hoc analysis of the MIDI model showed only differences between GE and Sola.

### 3.3 Reliability

**Fig. 4:**
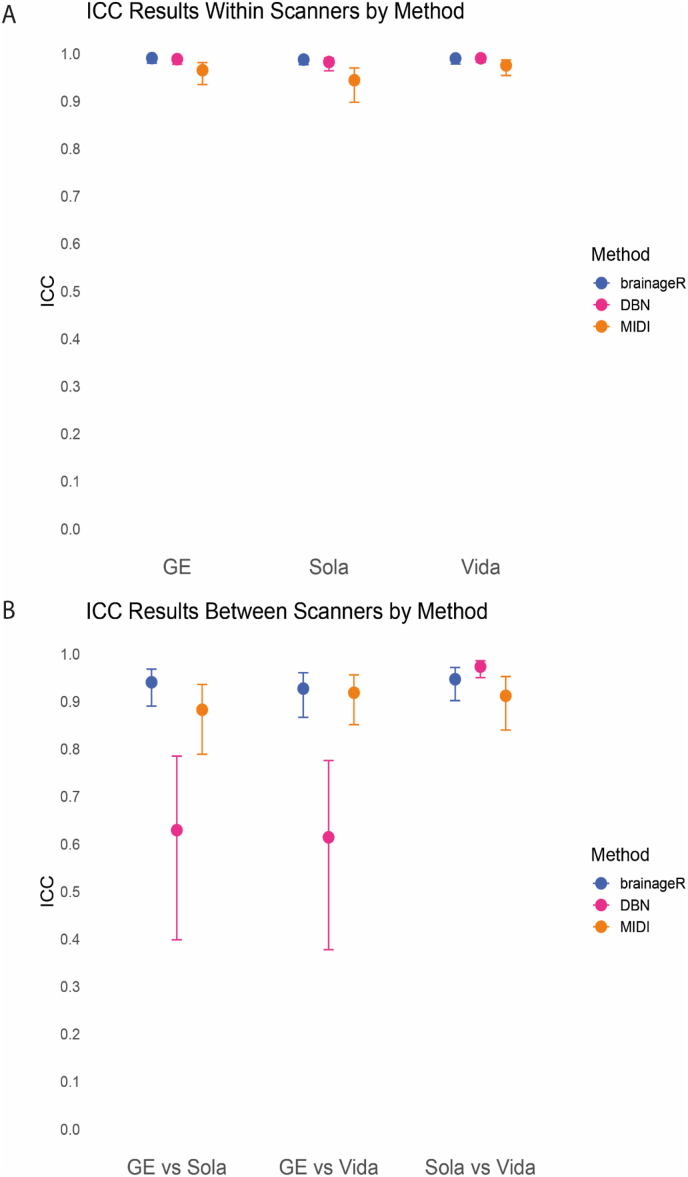
A) The ICC-AA with the 95% confidence interval for three brain age models for comparison of brain-PAD measurements with GE, Sola, and Vida. B) The ICC-C with the 95% confidence interval for three brain age models for comparison of brain-PAD measurements with GE vs Sola, GE vs Vida and Sola vs Vida.

**Fig. 5:**
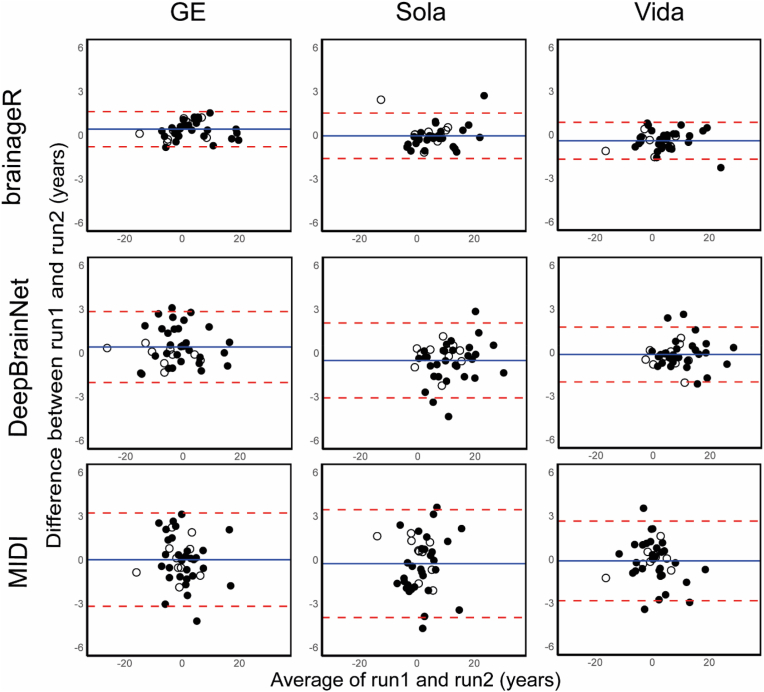
Bland-Altman plot for brain-PAD within-scanner comparison, where the difference between run 1 and run 2 of the scanners is plotted against the average of the two runs for three brain age models.

*Within*-scanner repeatability of brain-PAD estimates for each of the three scanners was excellent for all models (brainageR: ICC-AA = 0.99, DeepBrainNet: ICC-AA>0.98 and MIDI-model: ICC-AA>0.94) (Fig. 4). To visually assess the differences within-scanner, brain age of the first scan is plotted against the second scan in Fig. 5.

*Between*-scanner reproducibility of brain-PAD estimates was excellent between all pairwise scanner comparisons (ICC-C>0.93) for brainageR, between Sola-Vida for DeepBrainNet (ICC-C = 0.97) and between GE-Vida and Sola-Vida for the MIDI-model (ICC-C>0.91). Between-scanner reproducibility was good between GE-Sola (ICC-C = 0.88) for the MIDI-model. Between-scanner reproducibility for DeepBrainNet was moderate between GE and Sola (ICC-C = 0.63) and GE vs Vida (ICC-C = 0.61).

The between-model reproducibility of brain-PAD estimates was moderate between all pairwise model comparison, for all scanners, with a range of ICC-C between 0.60 and 0.77 (Supplementary Figure 1).

### 3.4 Precision

**Fig. 6:**
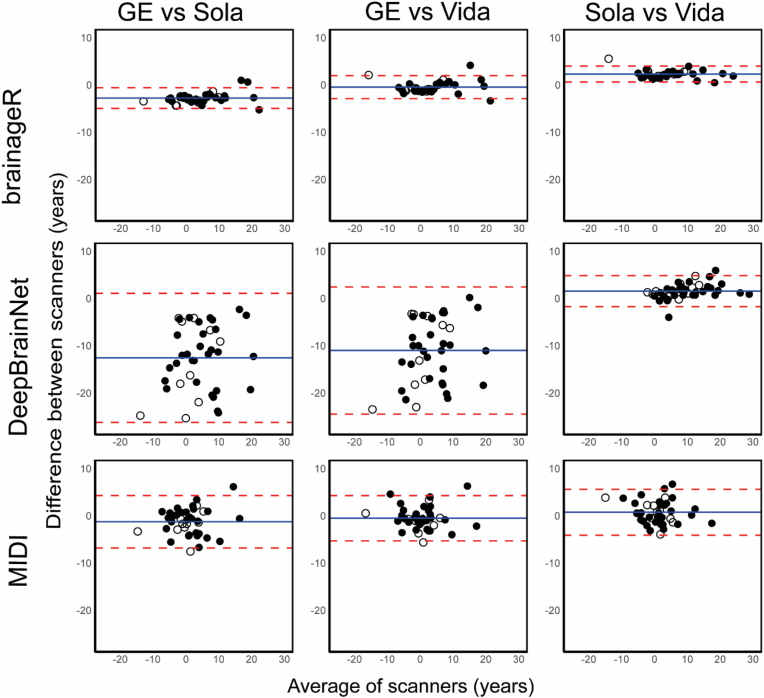
Bland-Altman plot for brain-PAD between-scanner comparison of brain-PAD determined on run 1 of each scanner.

The Bland-Altman plots for within-scanner brain-PAD do not show a systematic bias between the first and second run. The limit of agreement was smallest for brainageR and largest for the MIDI-model (Fig. 5). Between-scanner Bland-Altman plots showed a large window for limit of agreement for DeepBrainNet for scanner comparisons between GE and the 2 Siemens scanners. Brain-PAD predicted on the GE is also consistently lower than predictions on the Siemens scanners (Fig. 6). There was no evidence of bias for the other models and comparisons between-scanners. The Bland-Altman plot for all scanner comparisons with brainageR show the smallest window for the limit of agreement of all three models.

Within-scanner SDC for brainageR, DeepBrainNet and the MIDI-model were 2.07, 2.41 and 3.21 years, respectively. The SDC between-scanner was higher than within-scanner, for all brain age models. BrainageR and the MIDI-model demonstrated comparable results with an SDC estimate of 6.56 and 5.57 years, respectively. DeepBrainNet has 4 times as high an in SDC (SDC = 22.7 years) relative to the other two models. The SEM and the SDC of the brain-PAD is detailed in Table 4.

**Table 4:** Standard error of measurements (SEM) and smallest detectable change (SDC) in years for within or between-scanner brain PAD estimates for all three models.

Model	Within		Between	
	SEM (years)	SDC (years)	SEM (years)	SDC (years)
brainageR	0.75	2.07	2.37	6.56
DeepBrainNet	0.87	2.41	8.17	22.7
MIDI model	1.16	3.21	2.01	5.57

## 4 Discussion

The brain predicted age difference (brain-PAD) is a promising biomarker for prediction and monitoring of the disease course in neurodegenerative diseases, such as MS. The goal of this study was to quantify within-scanner repeatability and between-scanner reproducibility of these brain age estimates, for three publicly available brain age estimation models. These results allow informed decision making regarding the choice of brain age model given a particular population and study design. The study demonstrated excellent within-scanner repeatability for all brain age estimation models across a sample of HCs and pwMS. Between-scanner reproducibility was lower compared to within-scanner, depending on the brain age estimation model, implying that the effect of scanner type needs to be considered for each of the models assessed in this study. The RM-ANOVA measurements showed differences between brain-PAD between scanners for all three models. This shows that comparing brain-PAD predictions are increasingly challenging across scanners. DeepBrainNet demonstrated notably poor results when GE was compared to one of the Siemens scanners. The MIDI-model and brainageR exhibited higher between-scanner consistency, evident from intraclass correlation coefficient, overlapping confidence intervals and Bland-Altmann plots compared to DeepBrainNet. Between-model reproducibility was moderate for all model comparisons, on all scanners. This suggests that using different brain age models to compare results of brain age should be done with caution, as variations between models may influence the observed differences in brain age estimates. This pattern was consistent across all scanners, ensuring consistency in the choice of brain age model appears to be more important than using the same scanner.

A high ICC-value reflects strong reliability, indicating that subjects can be consistently distinguished from each other. This reliability can arise either because the differences between subjects are sufficiently large, or because the impact of other sources of variation (i.e., measurement error) is relatively minor (Mokkink et al., 2023). However, when there is considerable variation between subjects, measurement error can be substantial but still small enough in relation to the total variance to yield a high ICC. For this reason, the measurement error, quantified as the SEM and SDC, provides valuable additional insight into the precision of the measurement. Here, all scanners were combined in SDC-analyses to create a general measure for each brain age model, separately. The between-scanner SDC was higher than within-scanner, for all brain age models. The SDC from the MIDI-model was smaller compared to brainageR and DeepBrainNet. DeepBrainNet exhibited a substantial SDC of 22.7 years for pwMS, rendering brain-PAD estimates on different scanners inconclusive. The relatively high SDC for brain age prediction *between* scanners suggest that careful consideration is needed when comparing brain age predictions.

Scanner differences were present for all brain age models, these were dependent on the combination of brain age model, the scanner vendor, and the field strength. For brainageR, differences were presented between the Sola (1.5 T) and the other two scanners (3 T), for DeepBrainNet this was true for all scanner combinations, and for the MIDI-model we found differences between the GE and the Sola scanner. Images acquired at 1.5 T have a lower signal to noise ratio and contrast to noise compared to 3 T scanners (Chow et al., 2015), which could result in the lower reliability between 1.5 T vs 3 T scanners.

ICCs for consistency of DeepBrainNet indicated that scanner vendor is an important factor, as shown by the lower reliability between the GE and Siemens scanners when systematic differences are accounted for. This was also previously demonstrated for lesion segmentation algorithms using this data (Van Nederpelt et al., 2024b). Additionally, Bashyam et al. (Hobday et al., 2022) does not state the specific scanners on which the model was trained, it is possible that the training set could be lacking a similar scanner or protocol like the FSGPR sequence on GE scanners. As long as the primary hypothesis is not confounded by site (e.g., patient and control groups at each site), meaningful statistical results can still be derived when using multi-center datasets.

Brain-PAD was higher in pwMS than HC when using brainageR and DeepBrainNet, with medium effect sizes for all scanners, as assessed with the Cohen's d. The MIDI-model did not show differences between HCs and pwMS, on all scanners. This was reflected in the effect size, which was small for Sola and Vida and small to medium for GE. This observed pattern might be attributed to ‘regression towards the mean’, where extreme values in initial measurements tend to converge to the average (Bland and Altman, 1994). The use of the Cohen's d in this study provides a standardized measure of the magnitude of the differences observed between HC and pwMS. Effect sizes offer a standardized measure, which is particularly relevant when evaluating model performance in detecting disease-related effects on brain age. Models with low effect sizes may fail to capture meaningful brain-PAD differences, making it crucial to choose a model that reliably distinguishes MS from healthy controls. It is important to carefully select a brain age prediction model when investigating neurodegenerative diseases like MS.

### 4.1 Limitations and future work

The study highlighted the importance of consideration when multiple scanners are used in a study to determine brain-PAD, or when assessing longitudinal changes in brain-PAD. The excellent within-scanner reliability for all brain-PAD measurements are promising for implementation of brain-PAD in a clinical setting in a single center. This study had a relatively small sample size of 30 pwMS and 10 healthy controls which makes it difficult to determine reliable estimates of accuracy, specificity, sensitivity or AUROC for the brain age models. Nonetheless, since all participants were scanned twice on three scanners, this yields 240 MRI examinations and a unique dataset to investigate scanner differences.

In addition, the research brain MRI scans in this study are different from clinical MRI scans. These data consisted of 3D T1w MRI, where the clinical data usually consists of a combination of 2D and 3D T2-weighted data, particularly FLAIR. Adding to that, this study was conducted in one center, with local optimized protocols for the specific center. Most current brain age models are only available for 3D T1w scans, which are not part of the current recommended sequences for MRI in MS (Wattjes et al., 2021), making it difficult to implement brain-age estimates on clinical data as well. It is critical for future investigations to probe multimodal MRI for the prediction of brain-PAD.

Multi-center harmonization is a known issue in neuroimaging and statistical approaches are generally not feasible in clinical practice (Bland and Altman, 1994). However, recent literature showed promising results to mitigate the impact of MR scanners and conditions on the accuracy of brain age prediction (Dinsdale et al., 2021; Dular et al., 2024). Future work could elaborate on developing practical and standardized methods to account for scanner-related variability and increasing their usability in a clinical setting.

## 5 Conclusion

The findings in this study showed that while brain age estimates can reliably be measured when repeated on the same scanner, scanner differences persist, irrespective of the brain age prediction model. For implementation of brain-PAD in a clinical setting or a multicenter/multi-scanner study, the effect of scanner type needs to be considered for each of the three brain age models assessed in this study.

## CRediT authorship contribution statement

**Lonneke Bos:** Writing – original draft, Methodology, Formal analysis, Conceptualization. **David R. van Nederpelt:** Writing – original draft, Methodology, Formal analysis, Conceptualization. **J.H. Cole:** Writing – review & editing, Methodology. **E.M.M. Strijbis:** Writing – review & editing. **B. Moraal:** Writing – review & editing. **J.P.A. Kuijer:** Writing – review & editing. **B.M.J. Uitdehaag:** Writing – review & editing, Supervision. **F. Barkhof:** Writing – review & editing, Supervision. **A.M. Wink:** Writing – review & editing, Supervision. **H. Vrenken:** Writing – review & editing, Supervision. **B. Jasperse:** Writing – review & editing, Supervision.

## Declaration of competing interest

The authors declare the following financial interests/personal relationships which may be considered as potential competing interests: **JHC** is on the advisory board of BrainKey and Claritas HealthTech PTE. **BMJU** reports research support and/or consultancy fees from Immunic Therapeutics. **FB** is a steering committee or Data Safety Monitoring Board member for Biogen, Merck, ATRI/ACTC and Prothena. Consultant for Roche, Celltrion, Rewind Therapeutics, Merck, IXICO, Jansen, Combinostics. Research agreements with Merck, Biogen, GE Healthcare, Roche. Co-founder and shareholder of Queen Square Analytics LTD. **HV** has received research support from 10.13039/100004334Merck, 10.13039/100004336Novartis, 10.13039/100004319Pfizer, and Teva, consulting fees from Merck, and speaker honoraria from Novartis; all funds were paid to his institution. **LB**, **DRvN**, **EMMS**, **BM**, **JPAK**, **AMW and BJ** report no disclosures.

## Data Availability

The authors do not have permission to share data.

## References

[bib1] Amiri H., de Sitter A., Bendfeldt K., Battaglini M., Wheeler-Kingshott C.A.G., Calabrese M. (2018). Urgent challenges in quantification and interpretation of brain grey matter atrophy in individual MS patients using MRI. Neuroimage: Clin..

[bib2] Ashburner J., Barnes G., Chen C.-C., Daunizeau J., Flandin G., Friston K. (2014).

[bib3] Bacas E., Kahhalé I., Raamana P.R., Pablo J.B., Anand A.S., Hanson J.L. (2023). Probing multiple algorithms to calculate brain age: examining reliability, relations with demographics, and predictive power. Hum. Brain Mapp..

[bib4] Bashyam V.M., Erus G., Doshi J., Habes M., Nasrallah I.M., Truelove-Hill M. (2020). MRI signatures of brain age and disease over the lifespan based on a deep brain network and 14 468 individuals worldwide. Brain.

[bib5] Biondo F., Jewell A., Pritchard M., Aarsland D., Steves C.J., Mueller C., Cole J.H. (2022). Brain-age is associated with progression to dementia in memory clinic patients. Neuroimage: Clin..

[bib6] Bjartmar C., Kinkel R.P., Kidd G., Rudick R.A., Trapp B.D. (2001). Axonal loss in normal-appearing white matter in a patient with acute MS. Neurology.

[bib7] Bland J.M., Altman D.G. (1994). Some examples of regression towards the mean. BMJ Br. Med. J. (Clin. Res. Ed.).

[bib8] Cardoso M.J., Li W., Brown R., Ma N., Kerfoot E., Wang Y. (2022).

[bib9] Chow N., Hwang K.S., Hurtz S., Green A.E., Somme J.H., Thompson P.M. (2015). Comparing 3T and 1.5 T MRI for mapping hippocampal atrophy in the Alzheimer's disease neuroimaging initiative. Am. J. Neuroradiol..

[bib10] Clausen A.N., Fercho K.A., Monsour M., Disner S., Salminen L., Haswell C.C. (2022). Assessment of brain age in posttraumatic stress disorder: findings from the ENIGMA PTSD and brain age working groups. Brain. behav..

[bib11] Cole J.H., Ritchie S.J., Bastin M.E., Hernández V., Muñoz Maniega S., Royle N. (2018). Brain age predicts mortality. Mol. Psychiatr..

[bib12] Cole J., Raffel J., Friede T., Eshaghi A., Brownlee W., Chard D. (2019). Accelerated brain ageing and disability in multiple sclerosis. bioRxiv.

[bib13] Cole J.H., Raffel J., Friede T., Eshaghi A., Brownlee W.J., Chard D. (2020). Longitudinal assessment of multiple sclerosis with the brain‐age paradigm. Ann. Neurol..

[bib14] Compston A., Winedl H., Kieseier B. (2008). Coles. Multiple sclerosis. Lancet.

[bib15] De Vet H.C., Terwee C.B., Mokkink L.B., Knol D.L. (2011).

[bib16] Dinsdale N.K., Jenkinson M., Namburete A.I. (2021). Deep learning-based unlearning of dataset bias for MRI harmonisation and confound removal. Neuroimage.

[bib17] Dular L., Pernuš F., Špiclin Ž., Initiative AsDN (2024). Extensive T1-weighted MRI preprocessing improves generalizability of deep brain age prediction models. Comput. Biol. Med..

[bib18] Fritz C.O., Morris P.E., Richler J.J. (2012). Effect size estimates: current use, calculations, and interpretation. J. Exp. Psychol. Gen..

[bib19] Hobday H., Cole J.H., Stanyard R.A., Daws R.E., Giampietro V., O'Daly O. (2022). Tissue volume estimation and age prediction using rapid structural brain scans. Sci. Rep..

[bib20] Høgestøl E.A., Kaufmann T., Nygaard G.O., Beyer M.K., Sowa P., Nordvik J.E. (2019). Cross-sectional and longitudinal MRI brain scans reveal accelerated brain aging in multiple sclerosis. Front. Neurol..

[bib21] Isensee F., Schell M., Pflueger I., Brugnara G., Bonekamp D., Neuberger U. (2019). Automated brain extraction of multisequence MRI using artificial neural networks. Hum. Brain Mapp..

[bib22] Jenkinson M., Smith S. (2001). A global optimisation method for robust affine registration of brain images. Med. Image Anal..

[bib23] Jenkinson M., Bannister P., Brady M., Smith S. (2002). Improved optimization for the robust and accurate linear registration and motion correction of brain images. Neuroimage.

[bib24] Karatzoglou A., Smola A., Hornik K., Karatzoglou M.A. (2019).

[bib25] Kaufmann T., van der Meer D., Doan N.T., Schwarz E., Lund M.J., Agartz I. (2019). Common brain disorders are associated with heritable patterns of apparent aging of the brain. Nat. Neurosci..

[bib26] Koo T.K., Li M.Y. (2016). A guideline of selecting and reporting intraclass correlation coefficients for reliability research. J. chiropr. med..

[bib27] Liu Y., Duan Y., Huang J., Ren Z., Liu Z., Dong H. (2018). Different patterns of longitudinal brain and spinal cord changes and their associations with disability progression in NMO and MS. Eur. Radiol..

[bib28] Mokkink L.B., Eekhout I., Boers M., van der Vleuten C.P., de Vet H.C. (2023). Studies on reliability and measurement error of measurements in medicine–from design to statistics explained for medical researchers. Patient Relat. Outcome Meas..

[bib29] Sullivan G.M., Feinn R. (2012). Using effect size—or why the P value is not enough. J. grad. med. educat..

[bib30] Thompson A.J., Banwell B.L., Barkhof F., Carroll W.M., Coetzee T., Comi G. (2018). Diagnosis of multiple sclerosis: 2017 revisions of the McDonald criteria. Lancet Neurol..

[bib31] Van Nederpelt D.R., Amiri H., Brouwer I., Noteboom S., Mokkink L.B., Barkhof F. (2023). Reliability of brain atrophy measurements in multiple sclerosis using MRI: an assessment of six freely available software packages for cross-sectional analyses. Neuroradiology.

[bib32] Van Nederpelt D., Vrenken H., Strijbis E., Barkhof F., Kuijer J. (2024). Amsterdam Univeristy Medical C.

[bib33] Van Nederpelt D.R., Pontillo G., Barrantes-Cepas M., Brouwer I., Strijbis E.M.M., Schoonheim M.M. (2024). Scanner-specific optimisation of automated lesion segmentation in MS. Neuroimage: Clin..

[bib34] Wattjes M.P., Ciccarelli O., Reich D.S., Banwell B., de Stefano N., Enzinger C. (2021). MAGNIMS–CMSC–NAIMS consensus recommendations on the use of MRI in patients with multiple sclerosis. Lancet Neurol..

[bib35] Wood D.A., Kafiabadi S., Al Busaidi A., Guilhem E., Montvila A., Lynch J. (2022). Accurate brain‐age models for routine clinical MRI examinations. Neuroimage.

