# Supplementary material for: Repeatability and reproducibility of brain age estimates in multiple sclerosis for three publicly available models
Source: Neuroimage Rep. 2025 Mar 21;5(2):100252. doi: 10.1016/j.ynirp.2025.100252 (PMC12172926; doi:10.1016/j.ynirp.2025.100252)
Supplement: Multimedia component 1 [file mmc1.docx]

***Supplementary Table 1*** *Mean absolute error and corresponding standard deviation in years of brain PAD estimates of the 10 HCs for each scanner and for all three models.*

| **Model** | **GE** MAE ± SD (years) | **Sola** MAE ± SD (years) | **Vida** MAE ± SD (years) |
| --- | --- | --- | --- |
| brainageR | 5.6 ± 7.1 | 5.6 ± 6.7 | 5.3 ± 7.3 |
| DeepBrainNet | 8.2 ± 9.3 | 7.7 ± 6.1 | 6.4 ± 5.3 |
| MIDI model | 4.2 ± 6.1 | 3.4 ± 5.3 | 4.0 ± 6.5 |

***
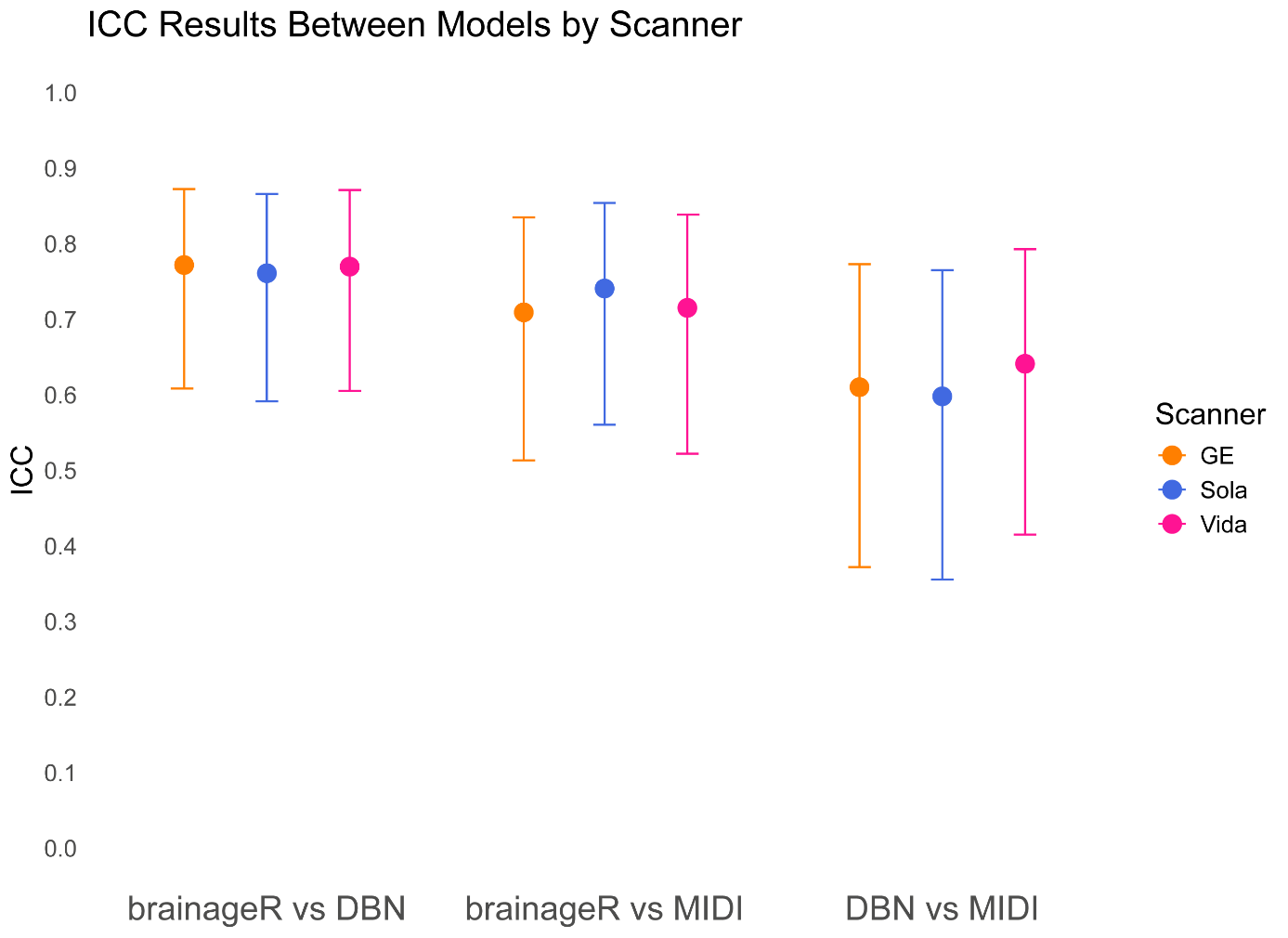
***

***Supplementary figure 1*** *The ICC-C with the 95% confidence interval for the three brain age models for comparison of brain-PAD measurements with brainageR vs DeepBrainNet, brainageR vs the MIDI-model, and DeepBrainNet vs the MIDI-model.*
